# α-Synuclein Levels in Blood Plasma from *LRRK2* Mutation Carriers

**DOI:** 10.1371/journal.pone.0052312

**Published:** 2012-12-27

**Authors:** Ana Gorostidi, Alberto Bergareche, Javier Ruiz-Martínez, José F. Martí-Massó, María Cruz, Shiji Varghese, Mohamed M. Qureshi, Fatimah Alzahmi, Abdulmonem Al-Hayani, Adolfo López de Munáin, Omar M.A. El-Agnaf

**Affiliations:** 1 Biodonostia Research Institute, Neurosciences area, Donostia, Gipuzkoa, Spain; 2 Hospital Donostia, Department of Neurology, Movement Disorders Unit, Donostia, Gipuzkoa, Spain; 3 Centro de investigación biomédica en Red para enfermedades Neurodegenerativas (CIBERNED), Carlos III Health Institute, Madrid, Spain; 4 Ikerbasque Basque Fundation for Science, Bilbao, Bizkaia, Spain; 5 Department of Biochemistry, Faculty of Medicine and Health Sciences, United Arab Emirates University, Al Ain, United Arab Emirates; 6 Department of Anatomy, Faculty of Medicine, King Abdulaziz University, Jeddah, Saudi Arabia; 7 Faculty of Medicine, King Abdulaziz University, Jeddah, Saudi Arabia; Centre Hospitalier Universitaire Vaudois (CHUV), Switzerland

## Abstract

The diagnosis of Parkinson’s disease (PD) remains primarily a clinical issue, based mainly on phenotypic patterns. The identification of biomarkers capable of permitting the preclinical detection of PD is critically needed. α-Synuclein is a key protein in PD, with missense and multiplication mutations in the gene encoding α-synuclein (*SNCA*) having been reported in familial cases of PD, and accumulation of the protein identified in Lewy bodies (LBs) and Lewy neurites (LNs) in affected brain regions. With the objective of validating the use of α-synuclein as a clinical or progressive biomarker in an accessible tissue, we used an enzyme-linked immunosorbent assay (ELISA) to measure α-synuclein levels in the peripheral blood plasma of idiopathic PD and *LRRK2* mutation carrier patients and compared our findings with healthy control subjects. Compared to healthy controls, we found a significant decrease in plasma total α-synuclein levels in idiopathic PD (iPD) patients (n = 134, *p* = 0.010). However, the reduction was less significant in patients who were *LRRK2* mutation carriers (n = 32, *p* = 0.133). This lack of significance could be due to the small number of individuals employed in this group. No predictive value of total α-synuclein in the diagnosis of PD was found in a receiver operating characteristic (ROC) curve analysis. Although this is a pilot study requiring corroboration on a larger cohort of patients, our results highlight the possible use of plasma α-synuclein as a biomarker for PD.

## Introduction

While Parkinson’s disease (PD) is the second most common neurodegenerative disease in humans, its etiology nevertheless remains largely unknown. The diagnosis of PD remains a clinical entity based on the presence of the cardinal motor signs. In addition, PD can be misdiagnosed for other forms of parkinsonism, even by experienced clinicians, especially in the early stages of the disease [1]. Therefore, reliable diagnostic markers would be valuable even in the pre-motor stage of the disease, particularly if disease modifying agents become available.

Although most PD patients have the idiopathic form of the disease (iPD), familial PD cases have been widely reported. PD associated with *LRRK2* mutations is the most common known genetic cause of autosomal dominant PD [2]–[4]. These cases commonly have a late onset and a typical clinical picture of iPD. The most frequent *LRRK2* mutation, G2019S, has been identified throughout the world, while others, like R1441G, show a more geographically specific localization, mainly in northern Spain [5]–[8].

The loss of dopaminergic neurons is a constant feature in every form of PD. Lewy bodies (LBs) and Lewy neurites (LNs) immunoreactive for α-synuclein constitute the neuropathological hallmark of iPD [9], although this finding is not universal in PD patients with the *LRRK2* mutation [10]. α-Synuclein misfolding and aggregation in the dopaminergic cells are considered to be pivotal factors in the degeneration process [11]. Actually, missense and multiplication mutations in *SNCA* are associated with familial PD and the formation of LBs and LNs [12]. The central nervous system has been proposed as the source of α-synuclein, and neurons are thought to release α-synuclein which is able to enter the cerebrospinal fluid (CSF) [13], [14], and α-synuclein has also been detected in blood plasma [13]. Recent studies have confirmed the presence of α-synuclein in such extracellular fluids [15]–[19]. Although α-synuclein in the CSF has been proposed as a biomarker of PD, relatively few studies have addressed the issue of what levels of α-synuclein are present in human plasma [16]–[19]. Data from these studies have been difficult to interpret, suggesting that more sensitive, standardized, and well-characterized assays of larger cohorts are required, as pointed out previously by Mollenhauer and colleagues [20].

It has been hypothesized that early aggregates or “soluble oligomers” of synuclein are the pathogenic species that lead to neuronal death and neurodegeneration rather than the insoluble late aggregates “amyloid fibril” [21], [22]. In this sense, increased levels of soluble α-synuclein oligomers have been identified in the plasma tissue and post mortem brain homogenates of PD patients [23]–[25]. In the present study we measured both the total and oligomeric forms of α-synuclein in blood plasma of patients with iPD and *LRRK2* forms of PD with a view to determine if differences exist between these two groups and healthy controls.

## Materials and Methods

### Subjects

Patients with PD were recruited from the Movement Disorders Unit of the Hospital Donostia (MDUD, Hospital Universitario Donostia, San Sebastian, Spain). Healthy controls were recruited from among the spouses of patients in the MDUD. PD was diagnosed according to the Gelb criteria by neurologists specialized in movement disorders [26]. Patients underwent a physical examination and completed a clinical questionnaire to provide details of demographic and clinical features of their condition. The clinical severity of parkinsonism was assessed according to the Hoehn and Yahr (H&Y) scale. All subjects provided their written informed consent to participate in the study, which was approved by the local Ethical Board of the Hospital (Hospital Universitario Donostia).

### Blood Plasma Samples

Blood samples (10 mL) were obtained from all non-fasted patients and healthy controls by venous puncture between the hours of 10 a.m. and 1 p.m. Samples were collected in plastic tubes containing EDTA, and the plasma was then separated by centrifugation at 3000 rpm at 4°C for 20 min. Plasma was collected in 0.2 ml plastic tubes and stored at –80°C. The samples were thawed on ice just prior to analysis.

### Genetic Analysis

DNA was extracted from peripheral blood using standard laboratory procedures. All patients and control individuals were screened for both 4321C>G (R1441G) and 6055G>A (G2019S) mutations in the LRRK2 gene (these being the most prevalent *LRRK2* mutations). Single nucleotide polymorphism genotyping was also performed using TaqMan chemistry on an ABI7300 instrument (Applied Biosystems, Foster City, CA) according to manufacturer’s instructions.

### Measurements of Total α-synuclein Levels in Plasma

Plasma total α-synuclein was measured using a sandwich ELISA assay as described previously [27], with some modifications aimed at improving sensitivity. Briefly, an anti-human α-synuclein monoclonal antibody 211 (mAb-211; Santa Cruz Biotechnology, USA) was used for capturing, and an anti-human α-synuclein polyclonal antibody (FL-140; Santa Cruz Biotechnology, USA) was used for antigen detection with a horseradish peroxidase (HRP)-linked chemiluminescence assay. The ELISA plate (Nunc Maxisorb, NUNC, Denmark) was coated for overnight incubation at 4°C with 1 µg/ml of mAb-211 (50 µl/well) in 200 mM NaHCO3, pH 9.6, and then washed 3× with (phosphate-buffered saline (PBS) containing 0.05% Tween 20 (PBST). After 2 hours of incubation 100 µl/well of blocking buffer (PBST containing 2.5% gelatin), 50µl of the plasma (diluted 1∶1 in PBST) was added to each well and incubated at 37°C for 2.5 hours. After incubation, the captured α-synuclein was mixed with FL-140 antibody (0.2 µg/ml, 50 µl/well), followed by incubation with 50 µl/well (1∶5,000-dilution in blocking buffer) of HRP-labeled goat anti-rabbit antibody (Jackson ImmunoResearch Laboratories, Inc., USA). Bound HRP activities were assayed by chemiluminescent reaction using 50 µl/well of an enhanced chemiluminescent substrate (SuperSignal ELISA Femto Maximum Sensitivity Substrate, Pierce Biotechnology, Rockford, USA). The chemiluminescence in relative light units was then immediately measured with a Victor^3^ 1420 (Wallac) microplate reader. The standard curve for the ELISA assay was constructed using 50 µl/well of recombinant human α-synuclein solution at different concentrations of the protein in blocking buffer. The relative concentration estimates of α-synuclein in CSF were calculated according to each standard curve. The intra- and inter-assay coefficients of variation were <10%. Samples were maintained on ice for all ELISA assays, with the assays performed on sample aliquots that had been thawed once.

### Measurements of Oligomeric α-synuclein Levels in Plasma

A 384-well ELISA microplate was coated by overnight incubation at 4°C with 1 µg/ml of mAb Syn211 in 200 mm NaHCO_3_, pH 9.6 (50 µl/well). The plate was washed with PBST and incubated with 100 µl/well of blocking buffer for 2 hours at 37°C. After washing, 50 µl of each plasma sample (diluted 1∶1 in PBST) were added to separate wells and the plate incubated at 37°C for a further 3 hours. Biotinylated Syn211 diluted to 0.5 µg/ml in blocking buffer was added and incubated at 37°C for 2 hours. The plate was washed with PBST and then incubated for 1 hour at 37°C with 50 µl/well of ExtrAvidin-Peroxidase (Sigma-Aldrich, Dorset, UK). The plate was washed again with PBST and incubated with 50 µl/well of an enhanced chemiluminescent substrate (SuperSignal ELISA Femto, Pierce Biotechnology, Rockford, IL) with 50 µl/well of the enhanced chemiluminescent substrate, after which chemiluminescence in relative light units was immediately measured. For both immunoassays, the samples were screened in a blind fashion and were randomly tested. The case and control samples were run in one plate to avoid plate-to-plate variations, and results were confirmed with at least two independent experiments.

### Statistical Analyses

Demographic and clinic variables were analyzed with ANOVA and Chi-square test. Differences between PD patients and control groups were compared using the Mann-Whitney U test. The level of significance was set at *p*<0.05. Correlation analyses were conducted using the Spearman simple correlation and Mann-Whitney U test to assess the potential relationship between α-synuclein expression and epidemiologic and clinical variables. Receiver operating characteristics (ROC) were analyzed to assess the most appropriate cut-off values for the levels of α-synuclein in the distinction between PD and control groups. General data is expressed as the mean ± the standard deviation (SD). All analyses were carried out using SPSS 16.0 software (SPSS Inc. Chicago, IL) for Windows XP (Microsoft, US).

## Results

### Clinical Features of Subjects

Plasma was obtained from 166 patients with PD and 109 healthy control subjects. Patients were classified as *LRRK2* mutation carriers (n = 32) or non-mutation carriers (iPD) (n = 134) according to both most prevalent *LRRK2* screened mutations (G2019S, n = 4 and R1441G, n = 28). Demographic features of the studied groups are summarized in Table 1.

**Table 1:** Demographic data of the samples.

	*Control*	*Patients*		*p*
		iPD	LRRk2 mutPD	
**Number**	109	134	32	
**Age at study**	69.9±16.7	69.0±10.6	68.0±10.1	0.139^a^
**Males % (n)**	39.5% (42)	57.4% (77)	40.6% (13)	0.013^b^
**Age at onset** **of PD**	NA	62.8±10.7	60.9±8.7	0.273^a^
**Disease** **duration (years)**	NA	6.2±5.3	7.5±6.3	0.217^a^
**Disease severity (H&Y score)**	NA	2.02±0.76	2.09±0.78	0.341 a

NA: not applicable.

Participants are grouped as healthy controls (Control), LRRK2 mutation carrier Parkinson’s disease patients (LRRK2 mutPD) and non-carrier or idiopathic Parkinson’s disease patients (iPD).

* The level of significance was set at *p*<0.05.

a Anova test.

b Chi-square test.

Patients and controls did not differ in age (*p* = 0.139; Table 1), although the proportion of males and females did differ between groups (*p* = 0.013). Disease severity information was available for 137/166 (83%) patients. There was no significant difference between the two groups of PD patients with respect to the duration, severity or age at onset of the disease.

### Levels of α-synuclein in Plasma from PD Patients and Control Individuals

**Figure 1:**
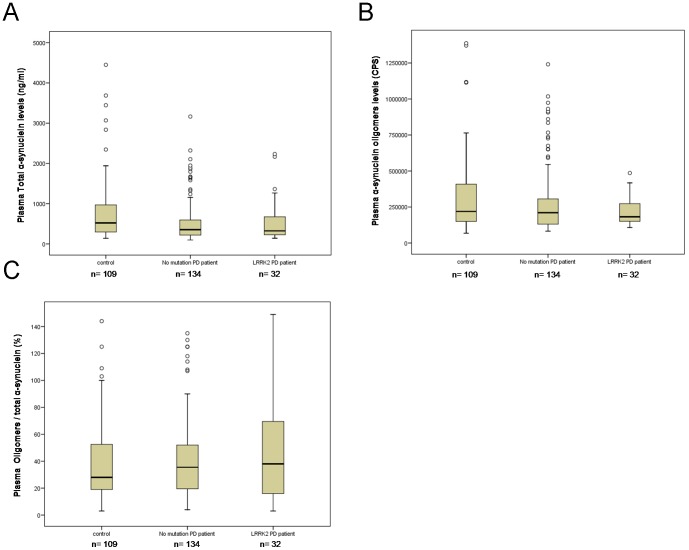
Box plot of plasma α-synuclein levels in Controls, iPD patients and *LRRK2* PD patients. **A)** Total α-synuclein (ng/ml). **B)** α-Synuclein oligomers in Counts Per Second (CPS). **C)** % Ratio Oligomers to total α-synuclein. Boxes show the minimum, maximum and median level for each group, together with the lower and upper quartile. Symbols (unfilled circles) outside the range represent outliers.

**Figure 2:**
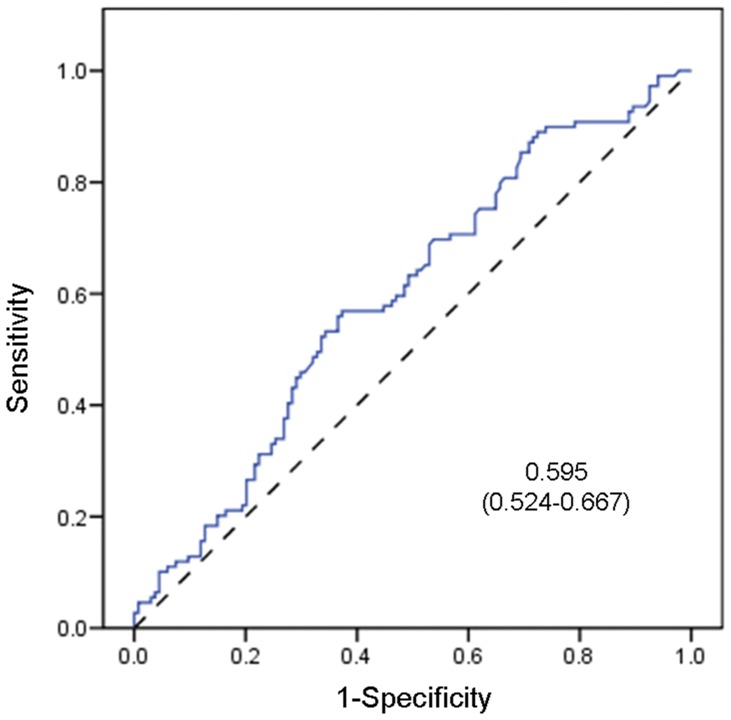
ROC curve for α-synuclein levels in iPD patients. A receiver operating characteristic (ROC) curve was generated for total α-synuclein in iPD patients. The dashed reference line represents the ROC curve for a test with no discriminatory ability. The area under the ROC curve (AUC) is displayed on the graph with the 95% confidence interval shown between the parentheses (0.524–0.667). The level of significance was set at *p*<0.05. No possible cutoff value was derived from the analysis.

Although there was a sizeable overlap in individual values between healthy controls and PD patients, we observed lower total α-synuclein plasma levels in iPD patients (n = 134, *p* = 0.010; see Table 2 and Figure 1). However, the reduction was less significant in patients who were *LRRK2* mutation carriers (n = 32, *p* = 0.133). The ROC curve analysis of the total α-synuclein levels did not discriminate between idiopathic patients and healthy controls (AUC = 0.595; 95% CI = 0.524–0667) (Table 2; Figure 2).

**Table 2:** α-synuclein values of the studied groups.

	*Control*	*Patients*		*p*			AUC	
		iPD	LRRk2 mutPD	iPD vs Contr	mutPD vs Contr	iPD vs mutPD	iPD vs Control	mutPD vs Control
**Number**	109	134	32					
**Total α-synuclein (ng/ml)**								
**Median (IQR)**	617.00 (307–1474)	386.5 (240.8–1068)	382.50 (240.7–1226)	0.010*	0.133	0.889	0.595 (0.524–0.667)	0.584 (0.464–0.705)
**α-synuclein Oligomers (CPS)**								
**Median (IQR)**	258275 (158861–537868)	230216.5 (143134.3–438763.3)	227894.5 (151752.5–295485.3)	0.143	0.224	0.958	0.555 (0.482–0.627)	0.580 (0.474–0.686)
**% Ratio Oligomers to total α-synuclein**								
**Median (IQR)**	29 (19–56)	36 (19.7–53)	38 (15.5–70.3)	0.346	0.492	0.798	0.465 (0.391–0.539)	0.472 (0.348–0.595)

CPS: counts per second.

Participants are grouped as healthy controls (Control), LRRK2 mutation carrier Parkinson’s disease patients (LRRK2 mutPD) and non-carrier or idiopathic Parkinson’s disease patients (iPD). Three different measurements of α-synuclein levels in plasma are shown for each group. Mann-Whitney *U* test results are specified for each comparison performed.

* The level of significance was set at *p*<0.05.

AUC: the Area Under the Curve for each analysis is shown with a 95% CI.

We did not observe any significant difference in the levels of α-synuclein oligomers between iPD patients and control groups (Table 2). Moreover, no differences between PD patients and controls were found with respect to the ratio of plasma α-synuclein oligomers to total α-synuclein (Figure 1).

### Correlation with Clinical Features of α-synuclein Levels in Human Plasma

Given the results of a previous report showing correlations between α-synuclein levels in the CSF with age, disease duration and severity [27], we also undertook a similar analysis and found no association between plasma α-synuclein levels and the parameters of age (*p* = 0.224 and R = 0.074), gender (*p* = 0.429; median in men = 414; median in women = 507.5), age at onset of the disease (*p* = 0.341 and R = −0.075) or disease duration (*p* = 0.903 and R = 0.010). Furthermore, no correlation with plasma α-synuclein levels was observed with respect to motor severity as measured by the H&Y scale (*p* = 0.458 and R = −0.064).

## Discussion

An important role has been attributed to α-synuclein in PD since the discovery of missense and multiplication mutations in *SNCA* associated with autosomal dominant familial PD [28], [29]. Furthermore, abnormal aggregates of α-synuclein protein were identified as the main components of LBs and LNs, the pathological hallmark of PD and dementia with Lewy bodies [9]. Since we reported the unexpected discovery of α-synuclein in the CSF and peripheral blood plasma [13], several groups have examined the potential use of α-synuclein as a putative biomarker for PD and other α-synucleinopathies, but the results have been inconclusive [16]–[19], [30]–[33]. Moreover, total and oligomeric forms of α-synuclein have been distinguished, with the latter seemingly more closely related to neuronal cell death and neurodegeneration, and therefore could potentially serve as a good biomarker for the early diagnosis of PD and monitoring of disease progression [20]–[25], [27].

Here we present the first case-control study of α-synuclein levels in the peripheral plasma of patients and controls, and provide data for both oligomeric and total α-synuclein levels. In addition, we have assessed both iPD patients and *LRRK2* mutation carrier PD patients as separate groups. Although total α-synuclein was significantly lower in iPD patients compared with controls (see Table 2; Figure 1), a similar reduction was also observed for the *LRRK2* patient group compared with controls (*p* = 0.133), however, the differences were not statistically significant (Table 2; Figure 1). This lack of significance could be due to the small number of individuals employed in this last-mentioned group. Alternatively, a different pathology associated with *LRRK2* mutations could explain the results. In this sense, while autopsy findings involving *LRRK2* results are not abundant, out of 28 reported cases [34]–[41], eight did not show LB inclusions [10], [36], [41]–[43]. We suggest however that the answer to this uncertainty could lie with the first possibility concerning group size, meaning that future studies assessing a larger number of cases with *LRRK2* mutations are needed to validate our findings.

Since the development of assays for measuring α-synuclein in biological fluids including blood plasma is only a relatively recent technological advance, few retrospective studies on this subject are currently available; moreover, these studies provide inconclusive and contradictory results [16]–[19], [30]–[33]. This could be due to differences in the assays and procedures employed by different laboratories, including the handling and storage of the plasma samples, and the use of different antibodies in the ELISA assays, thus ruling out the possibility for direct comparisons to be made of the results obtained. We previously showed that both anti-α-synuclein antibodies (FL-140 and mAb-211) used in our ELISA system are able to immunoprecipitate native α-synuclein from human plasma [13]. Importantly, the mAb-211 which we used as the capture antibody in our ELISA system is capable of recognizing only the full length α-synuclein protein in our biological samples. Some antibodies, however, can recognize both the full length and truncated forms of α-synuclein, making it necessary therefore to characterize antibody specificities carefully before they are employed in immunoassays designed to measure α-synuclein levels in biological fluids.

With respect to the plasma α-synuclein determination, we observed a significant reduction in levels in PD patients compared with controls (Figure 1). However, we are aware of the overlapping between PD and controls, and that our ELISA assay did not show sufficient sensitivity and specificity for the plasma α-synuclein levels to enable discrimination between the PD patient groups and healthy controls (AUC = 0.595; 95% CI = 0.524–0.667) (Table 2; Figure 2). These results might be at least partially explained by the small number of PD patients employed in our study. Other factors, such as age, disease duration and severity were not correlated with plasma α-synuclein levels in this cohort; however, the importance of these variables cannot be excluded in a more heterogeneous group. In this sense, it should be noted that the severity of the disease was relatively homogeneous as measured by the H&Y scale.

In our cohort we did not observe any significant difference in the levels of α-synuclein oligomers between iPD patients and control groups (Table 2). This is in disagreement with our previous reports [23], [27]. These contradictory findings might be explained by the small number of patients employed in our current study. Intriguingly, recent results of two independent studies have postulated that the native α-synuclein structure is not unfolded monomers but rather an α-helically folded tetramer that is stable and resistant to self-aggregation [44], [45]. Our current immunoassay for α-synuclein oligomers cannot distinguish between the toxic oligomers and the tetramers. This lack of specific oligomeric immunoassays for the toxic species of α-synuclein is an important limitation of our previous studies that have explored α-synuclein oligomers as a potential biomarker for PD. However, this research area is prone to speculation because a more recent study has challenged the α-synuclein tetramer hypothesis by demonstrating that unstructured monomers of α-synuclein were the predominant form in native conditions [46].

The identification of specific biomarkers for PD in peripheral blood plasma has advantages over the use of CSF biomarkers since obtaining plasma for such tests is far less invasive than obtaining CSF. On the other hand, plasma is a biochemically and physiologically more complex tissue to work with, as demonstrated in studies measuring Aβ42 protein levels in plasma as a biomarker for Alzheimer’s disease [47]. To this extent, a major limitation of plasma is that physiological modifiers of the levels of proteins like α-synuclein in plasma, such as environmental factors, pharmacotherapy, and circadian fluctuations in CSF and plasma exchange, are yet to be fully elucidated. Another aspect to be considered is that red blood cells contain α-synuclein. Barbour and colleagues suggested that α-synuclein levels in plasma and even in CSF may be artificially elevated by contamination with intact or lysed red blood cells due to their abundance and fragility [15], [30]. We should be aware of this putative bias and take it into consideration in any future studies to improve the outcome of this type of analysis. Larger scale studies are also needed to determine whether α-synuclein levels can be accurately measured in plasma and whether PD patients can be reliably distinguished from patients with α-synucleinopathies distinct from that of PD, and from healthy individuals. In spite of the unresolved issues surrounding the analytical tests for PD biomarkers, our results suggest that the accurate measurement of α-synuclein levels in plasma, if combined with other biomarkers (i.e. analytes from the proteome, transcriptome, metabolome, as well as neuroimaging, etc), could potentially serve as a valuable tool for improving the diagnostic accuracy of PD.
